# Characterization and Management of Adverse Reactions in Patients With Advanced Endometrial Cancer Receiving Lenvatinib Plus Pembrolizumab

**DOI:** 10.1093/oncolo/oyad201

**Published:** 2023-07-31

**Authors:** Nicoletta Colombo, Domenica Lorusso, Bradley J Monk, Brian Slomovitz, Kosei Hasegawa, Angélica Nogueira-Rodrigues, Melissa Zale, Chinyere E Okpara, Gianmaria Barresi, Jodi McKenzie, Vicky Makker

**Affiliations:** Gynecologic Oncology Department, European Institute of Oncology IRCCS, Milan, Italy; Department of Medicine and Surgery, University of Milan-Bicocca, Italy; Division of Gynecologic Oncology, Fondazione Policlinico Universitario Agostino Gemelli IRCCS and Catholic University of Sacred Heart, Rome, Italy; HonorHealth Research Institute, University of Arizona, Creighton University, Phoenix, AZ, USA; Division of Gynecologic Oncology, Mount Sinai Medical Center, Miami Beach, FL, USA; Department of Gynecologic Oncology, Saitama Medical University International Medical Center, Hidaka, Saitama, Japan; Universidade Federal de Minas Gerais (UFMG), Brazilian Group of Gynecologic Oncology (EVA), Grupo Oncoclínicas, DOM Oncologia, Brazil; Clinical Safety & Risk Management Late-Stage Oncology – Gynecologic Cancers, Merck & Co., Inc., Rahway, NJ, USA; Clinical Research, Eisai Ltd., Hatfield, UK; Merck & Co., Inc., Switzerland; Oncology Business Group, Eisai Inc., Nutley, NJ, USA; Department of Medicine, Memorial Sloan Kettering Cancer Center; Weill Cornell Medical Center, New York, NY, USA

**Keywords:** adverse reactions, lenvatinib, pembrolizumab, endometrial cancer

## Abstract

**Background:**

Lenvatinib plus pembrolizumab significantly improved efficacy compared with chemotherapy in patients with advanced endometrial cancer (aEC) regardless of microsatellite instability status or histologic subtype, who had disease progression following prior platinum-based therapy, in Study-309/KEYNOTE-775. The safety profile of the combination was generally consistent with that of each monotherapy drug and of the combination in patients with endometrial cancer and other solid tumors. Given the medical complexity of patients with aEC, this paper aims to characterize key adverse reactions (ARs) of the combination treatment and review management strategies, providing a guide for AR management to maximize anticancer benefits and minimize treatment discontinuation.

**Materials and Methods:**

In Study-309/KEYNOTE-775, patients received lenvatinib (20 mg orally once daily) plus pembrolizumab (200 mg intravenously every 3 weeks) or chemotherapy (doxorubicin or paclitaxel). The incidence and median time to the first onset of ARs, dose modifications, and concomitant medications are described. Key ARs characterized include hypothyroidism, hypertension, fatigue, diarrhea, musculoskeletal disorders, nausea, decreased appetite, vomiting, stomatitis, weight decreased, proteinuria, and palmar-plantar erythrodysesthesia syndrome.

**Results:**

As expected, the most common any-grade key ARs included: hypothyroidism, hypertension, fatigue, diarrhea, and musculoskeletal disorders. Grades 3-4 key ARs with incidence ≥10% included: hypertension, fatigue, and weight decreased. Key ARs first occurred within approximately 3 months of treatment initiation. AR management strategies consistent with the prescribing information and the study protocol are discussed.

**Conclusion:**

Successful AR management strategies for lenvatinib plus pembrolizumab include education of the patient and entire treatment team, preventative measures and close monitoring, and judicious use of dose modifications and concomitant medications.

**Clinicaltrials.gov ID:**

NCT03517449

## Teaser

The incidence of uterine cancer and its associated mortality rate are increasing. This article reports key adverse reactions in patients with advanced endometrial carcinoma who were treated with lenvatinib plus pembrolizumab and provides the clinician with comprehensive guidance for proactive adverse reaction management.

## Graphical Abstract

**Graphical Abstract:**
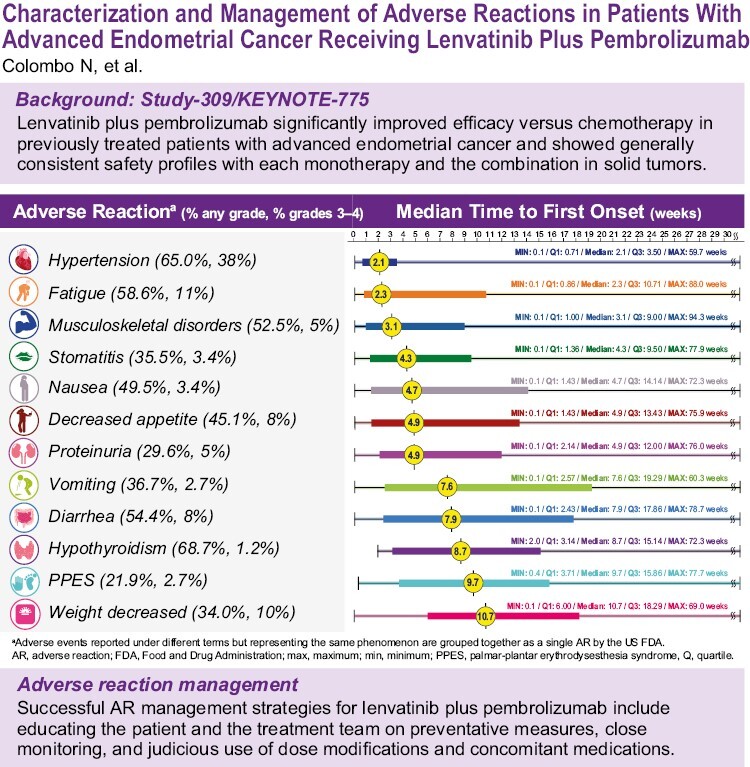

**Implications for Practice**

Lenvatinib plus pembrolizumab significantly improved efficacy compared with chemotherapy in patients with advanced endometrial carcinoma following at least 1 prior platinum-based therapy in any setting in Study-309/KEYNOTE-775. Key adverse reactions (ARs) associated with the combination include hypothyroidism, hypertension, fatigue, diarrhea, musculoskeletal disorders, nausea, decreased appetite, vomiting, stomatitis, weight decreased, proteinuria, and palmar-plantar erythrodysesthesia syndrome. AR management involves educating patients, family and caregivers, and the clinical team about mitigation strategies, close monitoring, dose modifications, and the use of concomitant medications. This report characterizes key ARs and summarizes management strategies in line with guidance from the prescribing information of each drug and the Study-309/KEYNOTE-775 protocol.

## Introduction

The incidence of uterine cancer and its associated mortality rate are increasing. Uterine cancer is projected to overtake colorectal cancer as the 3rd most common cancer by incidence (adjusted by average annual percent change) and the 4th most common cause of cancer death among women in the US.^1,2^ Globally, uterine corpus cancer is the 6th most diagnosed cancer in women, with over 417 000 new cases and 97 000 deaths in 2020.^3^ Among the 69% of patients who presented with localized disease in the US, the 5-year overall survival (OS) rate was 95% and the 5-year OS rate for distant disease was 18%.^4^ For most common cancers, survival rates have improved since the mid-1970s, with the notable exception of endometrial cancer (EC), reflecting a lack of significant treatment advances for this malignancy.^4^ Frontline carboplatin-paclitaxel chemotherapy is the standard-of-care first-line regimen based on the GOG0209 trial.^5,6^ The benefit of second-line chemotherapy is modest, with median progression-free survival (PFS) of approximately 4 months,^6^ underscoring the premise that previously treated advanced EC (aEC) is largely a chemotherapy-resistant disease. Hence, a tremendous unmet need still exists in patients with aEC for the development of improved therapeutics.

Lenvatinib, a tyrosine kinase inhibitor, plus pembrolizumab, an immune checkpoint inhibitor, significantly improved outcomes versus chemotherapy in patients with aEC in the Study-309/KEYNOTE-775 trial (Clinicaltrials.gov identifier: NCT03517449).^7^ Statistically significant and clinically meaningful improvements were seen in PFS, OS, and objective response rate (ORR) with lenvatinib plus pembrolizumab compared with chemotherapy in all patients and in the mismatch-repair proficient (pMMR) population, which were the prespecified populations for statistical testing. Additionally, the mismatch-repair deficient (dMMR) subgroup exhibited longer PFS, OS, and higher ORR with lenvatinib plus pembrolizumab compared with chemotherapy.^7^ Although results from post hoc analyses should be treated with caution, the treatment benefit of lenvatinib plus pembrolizumab versus chemotherapy in PFS and OS was observed across all histologies (including difficult-to-treat histologies), and was irrespective of prior (neo)adjuvant therapy and platinum-free interval from most-recent platinum-containing regimen (Supplementary Figs. S1–S3).^8^ Patients with 1 prior line of platinum therapy had more-favorable hazard ratios for OS and PFS than those with >1 prior lines of platinum therapy, supporting earlier use of lenvatinib plus pembrolizumab (Supplementary Fig. S3).^8^ Among patients treated with lenvatinib plus pembrolizumab, chemotherapy was the most common subsequent anticancer medication (Supplementary Fig. S4); patients continued to show clinically meaningful improvements in PFS on their next line of therapy compared with the control (Supplementary Fig. S5).^9^

Based on results from Study-309/KEYNOTE-775, lenvatinib plus pembrolizumab was approved in the US for the treatment of patients with advanced endometrial carcinoma that is mismatch repair proficient (pMMR), (as determined by a Food and Drug Administration-approved test), or not microsatellite instability-high, who have disease progression following prior systemic therapy in any setting, and who are not candidates for curative surgery or radiation.^10^ In Europe, the combination is approved for patients with advanced or recurrent endometrial carcinoma who have disease progression on or following prior treatment with a platinum-containing therapy in any setting and are not candidates for curative surgery or radiation.^11^

In Study-309/KEYNOTE-775, the safety profile of lenvatinib plus pembrolizumab was considered manageable and generally consistent with the established profiles of each monotherapy and of the combination in patients with EC and other solid tumor types.^7,10,12-14^ Treatment outcomes and tolerability in patients with aEC may additionally depend on factors including frailty, comorbidities, and age.^15^ The aim of this analysis is to review key adverse reactions (ARs) in patients with aEC who were treated with lenvatinib plus pembrolizumab in Study-309/KEYNOTE-775 to provide clinical teams with a comprehensive guide for proactive AR management, accessible to healthcare professionals with varying degrees of experience. We also review management strategies for ARs consistent with the prescribing information and study protocol, to maximize patient safety and support continuation of treatment, thus affording patients the best opportunity to benefit from the antitumor activity of this important therapeutic option. Finally, an iterative example of how these strategies can be implemented is provided in the context of a case study.

## Materials and Methods

### Patients and Study Design

The study design and other eligibility criteria have been published previously.^7^ Patients with aEC who had disease progression after 1 prior platinum-based chemotherapy were randomly assigned (1:1) to receive lenvatinib (starting dose of 20 mg orally once daily) and pembrolizumab (200 mg intravenously every 3 weeks) or chemotherapy (details in Supplementary Appendix).

### Adverse Reactions

**Figure 1.**
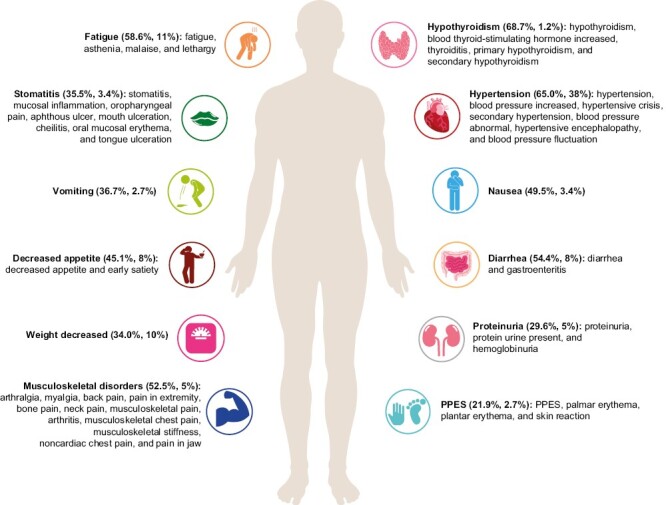
Preferred terms^a^ included in each key AR from Study-309/KEYNOTE-775 (safety analysis population). ^a^Preferred terms (% any grade, % grades 3-4) defined by the National Cancer Institute Common Terminology Criteria for Adverse Events, Version 4.0. PPES, palmar-plantar erythrodysesthesia syndrome. Template adapted from Powered Template https://poweredtemplate.com/

This analysis, at the data cutoff date of October 26, 2020, focused on the characterization and management of ARs (defined in the Supplementary Appendix), consistent with the prescribing information,^10,12^ in patients with previously treated aEC from Study-309/KEYNOTE-775 who were randomly assigned and treated with at least 1 dose of lenvatinib plus pembrolizumab. Preferred terms included in each key AR (defined in the Supplementary Appendix) are shown in Fig. 1. ARs could have occurred while receiving lenvatinib and/or pembrolizumab or within the protocol-defined follow-up period of approximately 30 days after the last dose of study treatment or before the initiation of a new anticancer treatment, whichever came first. Grading of ARs was performed according to Common Terminology Criteria for Adverse Events v4.03 (Supplementary Table S1).

## Results

### Patients

Of all patients enrolled, 411 were randomly assigned to receive lenvatinib plus pembrolizumab (406 patients received treatment).^7^ In the pMMR population, 346 patients were randomly assigned to receive lenvatinib plus pembrolizumab (342 patients received treatment).^7^ Patient disposition and baseline characteristics have been described previously.^7^ The median duration of lenvatinib plus pembrolizumab therapy was 231 days (range 1 to 817) among all patients.^7^

### Common and Key Adverse Reactions

**Figure 2.**
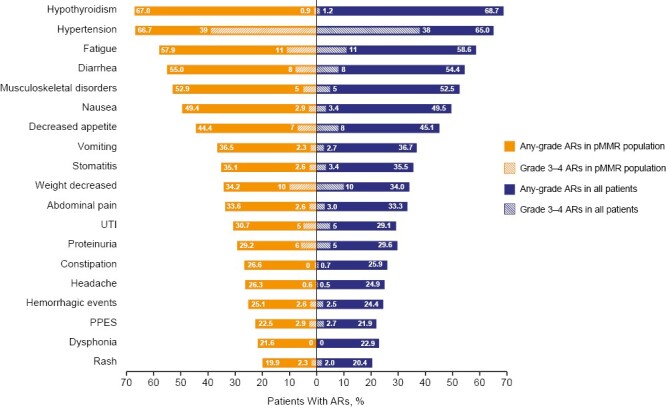
ARs with incidence >20% in all patients and in the pMMR population in the lenvatinib plus pembrolizumab arm of Study-309/KEYNOTE-775 (safety analysis population).^a^ All numbers included are percentages. No grade 5 events were observed among key ARs except for 1 event (0.2%) of grade 5 decreased appetite (not related to study drugs per investigator assessment). ^a^Grades 3-4 ARs (shaded bars) are a subset of any-grade ARs (solid bars). AR, adverse reaction; pMMR, mismatch repair proficient; PPES, palmar-plantar erythrodysesthesia syndrome; UTI, urinary tract infection.

ARs occurring in >50% of patients included hypothyroidism (all patients, 68.7%; pMMR, 67.0%), hypertension (all patients, 65.0%; pMMR, 66.7%), fatigue (all patients, 58.6%; pMMR, 57.9%), diarrhea (all patients, 54.4%; pMMR, 55.0%), and musculoskeletal disorders (all patients, 52.5%; pMMR, 52.9%; Fig. 2). Given the similar incidences of ARs between the all-patients and pMMR groups, data presented herein are for all patients who received at least 1 dose of study medication. Data regarding the pMMR population are included in the Supplementary Appendix for completeness.

**Figure 3.**
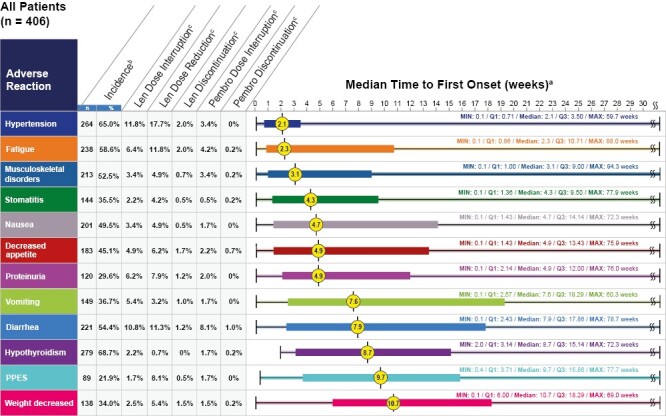
Median time to first onset^a^ of key ARs and dose management of patients in the lenvatinib plus pembrolizumab arm of Study-309/KEYNOTE-775 (safety analysis population). Preventative measures, monitoring, and management of ARs associated with lenvatinib and/or pembrolizumab during pretreatment and treatment phases are elaborated in Supplementary Fig. S7. ^a^Median time to first onset in patients who experienced the adverse reaction. ^b^All grades. ^c^Percentages of dose modifications and discontinuations were based on the safety analysis set. Len, lenvatinib; max, maximum; min, minimum; Pembro, pembrolizumab; PPES, palmar-plantar erythrodysesthesia syndrome; Q, quartile.

Key ARs (Fig. 1) characterized in our analysis include hypothyroidism, hypertension, fatigue, diarrhea, musculoskeletal disorders, nausea, decreased appetite, vomiting, stomatitis, weight decreased, proteinuria, and palmar-plantar erythrodysesthesia syndrome (PPES). When adjusted for exposure (Supplementary Appendix; Supplementary Table S2), the most frequent key ARs were diarrhea, hypertension, and musculoskeletal disorders. The incidences of grades 3-5 AR events are noted in Fig. 2. The median times to onset of key ARs and associated dose modifications are provided in Fig. 3^16^; corresponding pMMR population data are included in Supplementary Fig. S6.

#### General Management Strategies

Prior to beginning treatment, we recommend proactively training the entire clinical team to watch for common ARs and developing consistent management strategies, as this knowledge at all levels is critical for efficient management of ARs (Supplementary Fig. S7). Patient education to ensure optimal control of blood pressure (BP), nausea, bowel function, pain, oral intake, and any skin concerns prior to the initiation of lenvatinib plus pembrolizumab treatment is also important.

**Figure 4.**
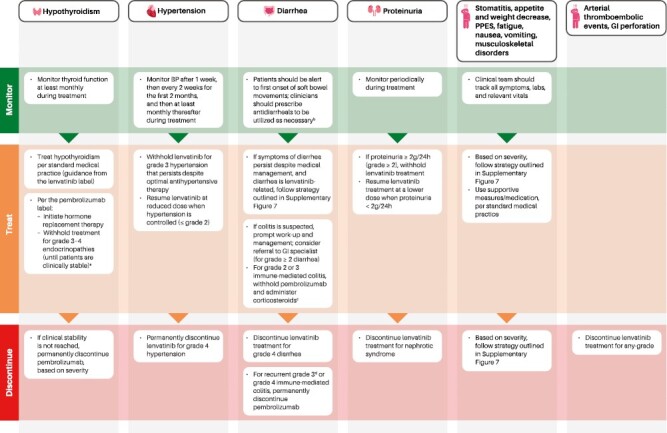
Management of select ARs during the treatment phase. For nausea, vomiting, hypertension, hypothyroidism, and/or diarrhea, optimal medical management is recommended prior to any lenvatinib interruption or dose reduction. Dose reductions of pembrolizumab are not recommended. ^a^Per the study protocol, pembrolizumab treatment could be continued for grades 2-4 hypothyroidism while initiating thyroid replacement hormones (eg, levothyroxine or liothyronine) per standard of care. ^b^The choice of antidiarrheal agent should be individualized to the participant’s clinical circumstances and follow standard medical practice. ^c^Resume pembrolizumab in patients with complete or partial resolution (grades 0 to 1) after corticosteroid taper. Permanently discontinue if no complete or partial resolution within 12 weeks of initiating steroids or inability to reduce prednisone to 10 mg per day or less (or equivalent) within 12 weeks of initiating steroids. ^d^Per the study protocol, pembrolizumab should be permanently discontinued for recurrent grade 3 or grade 4 immune-mediated colitis. AR, adverse reaction; BP, blood pressure; GI, gastrointestinal; PPES, palmar-plantar erythrodysesthesia syndrome; QD, every day.

The management strategy is to first determine whether the AR is related to lenvatinib or pembrolizumab, for instance by considering the timing or resolution of the event in relation to the administration of lenvatinib and pembrolizumab. For most key ARs (Supplementary Fig. S7; Fig. 4), as detailed in the lenvatinib prescribing information, lenvatinib is to be withheld for persistent or intolerable grade 2 or any grade 3 ARs, and treatment is to be discontinued for most grade 4 ARs.^10^ Upon resolution of the ARs to grade ≤1 or baseline, lenvatinib can be reduced progressively (to 14 mg, 10 mg, and 8 mg, each once daily)^10^ (Supplementary Fig. S7; Fig. 4). In Study-309/KEYNOTE-775, a further lenvatinib dose reduction to 4 mg was allowed with approval from the sponsor. Per the study protocol, patients were allowed to resume lenvatinib at a reduced dose level upon resolution of most ARs to tolerable grade 2 or grade ≤1, unless noted otherwise.^7^

Details regarding management of ARs related to pembrolizumab are included in the pembrolizumab prescribing information.^12^ Dose reduction for pembrolizumab is not recommended^12^ (Supplementary Fig. S7; Fig. 4). For most immune-mediated ARs (imARs) associated with pembrolizumab that are grade 2 or grade 3, treatment should be withheld and then resumed upon improvement to grade 0 or 1 after corticosteroid taper. Pembrolizumab should be permanently discontinued for life-threatening grade 4 imARs, recurrent severe (grade 3) imARs that require systemic immunosuppressive treatment, or if the imAR does not resolve within 12 weeks of initiating steroids or if corticosteroids cannot be reduced to ≤10 mg prednisone per day (or equivalent) within 12 weeks. Corticosteroid taper should be initiated when the imAR improves to grade 0 or 1 and tapering should continue over at least 4 weeks. For severe and life-threatening imARs, intravenous corticosteroids should be initiated first, before transitioning to oral steroids. Other immunosuppressive treatment should be initiated if imARs cannot be controlled by corticosteroids.^7,12^

For nausea, vomiting, hypertension, hypothyroidism, and/or diarrhea, optimal medical management is recommended prior to any lenvatinib interruption or dose reduction^7^ (Supplementary Fig. S7). Monitoring requirements for hypertension, diarrhea, proteinuria, and hypothyroidism are detailed in Supplementary Fig. S7 and the prescribing information.^10,12^ Specific management strategies including dose modifications for some of these key ARs with monitoring requirements before and/or during treatment are described in the sections below. Concomitant medications are also part of the AR management strategy per standard medical practice; associated data for common concomitant medications received by patients from Study-309/KEYNOTE-775 are provided (Table 1). Corresponding data for the pMMR population are presented in Supplementary Table S3.

**Table 1.** Summary of concomitant medications for the management of key adverse reactions in all patients from Study-309/KEYNOTE-775 (safety analysis population)

Adverse reaction Medications received^a^, *n*^b^ (%)	All patients; lenvatinib + pembrolizumab group (*n* = 406)
Hypothyroidism	
Patients with this AR	279 (100.0)
Patients who received ≥ 1 concomitant medication	216 (77.4)
Levothyroxine sodium	213 (76.3)
Hypertension	
Patients with this AR	264 (100.0)
Patients who received ≥ 1 concomitant medication	216 (81.8)
Amlodipine	80 (30.3)
Amlodipine besilate	49 (18.6)
Losartan	28 (10.6)
Captopril	21 (8.0)
Ramipril	20 (7.6)
Furosemide	18 (6.8)
Nifedipine	17 (6.4)
Hydrochlorothiazide	16 (6.1)
Lisinoprol	14 (5.3)
Fatigue	
Patients with this AR	238 (100.0)
Patients who received ≥ 1 concomitant medication	12 (5.0)
Dexamethasone	4 (1.7)
Diarrhea^c^	
Patients with this AR	221 (100.0)
Patients who received ≥ 1 concomitant medication	141 (63.8)
Loperamide hydrochloride	61 (27.6)
Loperamide	58 (26.2)
Musculoskeletal disorders	
Patients with this AR	213 (100.0)
Patients who received ≥ 1 concomitant medication	125 (58.7)
Paracetamol	59 (27.7)
Ibuprofen	23 (10.8)
Loxoprofen sodium	14 (6.6)
Prednisone	11 (5.2)
Nausea	
Patients with this AR	201 (100.0)
Patients who received ≥ 1 concomitant medication	131 (65.2)
Ondansetron	41 (20.4)
Metoclopramide hydrochloride	36 (17.9)
Metoclopramide	31 (15.4)
Prochlorperazine	16 (8.0)
Decreased appetite	
Patients with this AR	183 (100.0)
Patients who received ≥ 1 concomitant medication	42 (23.0)
Megestrol acetate	10 (5.5)
Vomiting	
Patients with this AR	149 (100.0)
Patients who received ≥ 1 concomitant medication	52 (34.9)
Metoclopramide	17 (11.4)
Ondansetron	16 (10.7)
Metoclopramide hydrochloride	13 (8.7)
Stomatitis	
Patients with this AR	144 (100.0)
Patients who received ≥ 1 concomitant medication	91 (63.2)
Nystatin	19 (13.2)
Dexamethasone	12 (8.3)
Sodium gualenate	10 (6.9)
Chlorhexidine gluconate	8 (5.6)
Lidocaine	8 (5.6)
Weight decreased	
Patients with this AR	138 (100.0)
Patients who received ≥ 1 concomitant medication	17 (12.3)
Nutrients nos	4 (2.9)
Proteinuria	
Patients with this AR	120 (100.0)
Patients who received ≥ 1 concomitant medication	5 (4.2)
Akritoin	1 (0.8)
Palmar-plantar erythrodysesthesia syndrome	
Patients with this AR	89 (100.0)
Patients who received ≥ 1 concomitant medication	62 (69.7)
Clobetasol propionate	15 (16.9)
Mucopolysaccharide polysulfuric acid ester	9 (10.1)
Urea	9 (10.1)
Heparinoid	6 (6.7)
Difluprednate	5 (5.6)

^a^Medications included are those received by ≥ 5% of patients *or* the most common concomitant medication received for the listed AR. Percentages are calculated based on the number of participants with the AR.

^b^Patients may have received > 1 medication to treat a specific AR.

^c^Diarrhea encompasses only diarrhea and gastroenteritis, and not colitis, which is immune-mediated and treated with steroids and other therapies.

AR, adverse reaction; nos, not otherwise specified.

#### Hypothyroidism

Hypothyroidism has been previously reported with both lenvatinib and pembrolizumab monotherapies.^10,12^ In Study-309/KEYNOTE-775, any-grade hypothyroidism occurred in 68.7% (*n* = 279) of patients and the median time to first onset was 8.7 weeks (Figs. 2 and 3). Most hypothyroidism events were of low-grade severity and were manageable with hormone replacement and without the need for dose modifications. Per the lenvatinib label, thyroid function of patients should be monitored prior to initiating treatment and at least monthly during treatment; hypothyroidism should be treated per standard medical practice (Supplementary Fig. S7; Fig. 4).^10^

Per the pembrolizumab label, hormone replacement therapy should be initiated for hypothyroidism, and pembrolizumab treatment should either be withheld for grades 3-4 endocrinopathies (until patients are clinically stable) or permanently discontinued, depending on severity.^12^ Per the study protocol, pembrolizumab treatment can be continued for grades 2–4 hypothyroidism while initiating thyroid replacement hormones, per standard of care.^7^

#### Hypertension

Any-grade hypertension occurred in 65.0% (*n* = 264) of patients and the median time to first onset was 2.1 weeks (Figs. 2 and 3).

BP should be optimized and controlled prior to starting treatment and monitored regularly during treatment^10^; ideally, patients should be as close to normotensive as possible. Patients with pre-existing hypertension should be on a stable dose of antihypertensive therapy for at least a week before starting the combination treatment. Per the study protocol, hypertension should be graded based only on BP measurements and not on the number of antihypertensive medications.^7^ Lenvatinib should be withheld for patients with grade 3 hypertension despite optimal antihypertensive therapy or in any instance where a patient is at imminent risk to develop a hypertensive crisis or if the patient has significant risk factors for severe complications of uncontrolled hypertension; treatment can be resumed at a reduced dose when controlled at grade 2 or lower severity.^10^ Lenvatinib should be permanently discontinued for grade 4 hypertension^10^ (Supplementary Fig. S7; Fig. 4).

#### Diarrhea

Any-grade diarrhea occurred in 54.4% (*n* = 221) of patients and the median time to first onset was 7.9 weeks (Figs. 2 and 3).

Prompt initiation of management of diarrhea is recommended.^10^ Patients should be alert to the first onset of soft bowel movements and should maintain adequate hydration with clear fluids (Supplementary Fig. 7). Clinicians should prescribe antidiarrheals to patients at the time of treatment initiation, to be utilized as needed. The choice of antidiarrheal agent should be individualized to the patient’s clinical circumstances and follow standard medical practice.^7^ Lenvatinib should be withheld and then resumed at a lower dose level or permanently discontinued based on severity of diarrhea (Supplementary Fig. 7).^10^

Pembrolizumab can cause immune-mediated colitis; hence, patients should be monitored for enterocolitis (diarrhea, abdominal pain, blood or mucus in stool with or without fever).^7,12^ In cases of suspected colitis, appropriate work-up including imaging and endoscopy should be initiated, medical management should be promptly initiated, and a gastrointestinal consult should be obtained (for grade ≥2 diarrhea), as applicable. For grades 2-3 immune-mediated diarrhea/colitis, pembrolizumab should be withheld and corticosteroids (initial dose of 1-2 mg/kg prednisone or equivalent, followed by taper) should be administered; for recurrent grade 3 or grade 4 immune-mediated diarrhea/colitis, pembrolizumab should be permanently discontinued (Fig. 4).^7,12^

#### Nausea and Vomiting

Any-grade nausea occurred in 49.5% (*n* = 201) of patients and the median time to first onset was 4.7 weeks (Figs. 2 and 3). Any-grade vomiting occurred in 36.7% (*n* = 149) of patients and the median time to first onset was 7.6 weeks (Figs. 2 and 3). Clinicians should prescribe antiemetics at the time of treatment initiation, to be used as needed, and should treat nausea and vomiting before dose-reducing lenvatinib.

#### Proteinuria

Any-grade proteinuria occurred in 29.6% (*n* = 120) of patients and the median time to first onset was 4.9 weeks (Figs. 2 and 3).

Monitoring proteinuria prior to initiation of treatment and periodic monitoring during treatment are recommended^10^ (Supplementary Fig. S7; Fig. 4). Lenvatinib should be withheld for ≥2g of proteinuria/24 h and then resumed at a lower dose when proteinuria is <2g/24 h.^11^ Treatment should be discontinued for nephrotic syndrome^10^ (Fig. 4).

#### Overlapping Toxicities

In Study-309/KEYNOTE-775, the safety profile observed for lenvatinib plus pembrolizumab was generally consistent with the known safety profiles of lenvatinib and pembrolizumab when used as monotherapies and as a combination in patients with endometrial cancer and other solid tumors, with no new safety signals identified.^7,10,12-14^

In instances where both agents could cause an AR (eg, diarrhea, liver enzyme elevations), the timing of AR onset and AR resolution with treatment interruption can be evaluated in the context of the shorter half-life of lenvatinib. If dose interruption of lenvatinib does not lead to clinical improvement, an imAR may be considered (Supplementary Fig. 8). Severe ARs may sometimes require interruption of both drugs and prompt initiation of treatment.

In cases where diarrhea persists despite medical management, lenvatinib should be withheld and resumed at a lower dose or discontinued, based on severity. If colitis is suspected, adequate evaluation should be used to confirm etiology or exclude other causes such as bacterial or viral infections. If determined to be immune-mediated, management guidelines should be followed. In cases of elevated liver enzymes, other offending agents (such as paracetamol), infection (eg, viral hepatitis), or metastatic disease should be ruled out, while following advice from the prescribing information.

## Case Study Vignette

**Figure 5.**
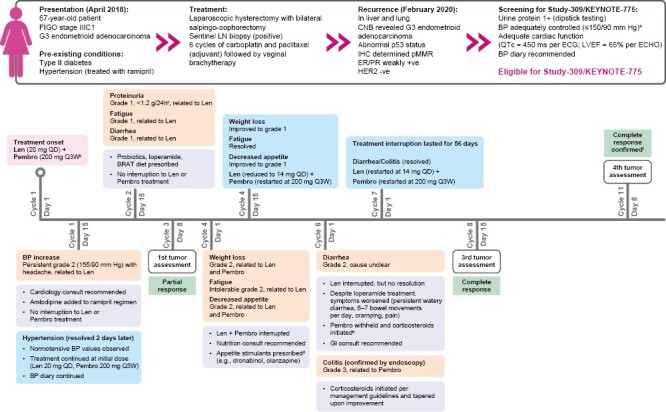
Case study vignette. ^a^BP was adequately controlled (by ramipril for at least 1 week prior to treatment) per protocol requirements (≤150/90 mmHg). ^b^Treatment began in March 2020. ^c^Urine dipstick testing showed 2+ proteinuria, which was confirmed as grade 1 following a 24-h urine collection (<1.2 g/24 h). ^d^Small, frequent, and high-calorie meals (full-fat milks, yogurt, cheeses, peanut butter, avocado, etc.) were suggested, along with nutritional shakes. ^e^Prednisone initial dose 1 mg/kg, followed by taper once symptoms improved. ^f^The patient completed 35 cycles of treatment with pembrolizumab and continued treatment with lenvatinib. BP, blood pressure; BRAT, bananas, rice, applesauce, and toast; CNB, core needle biospy; ECG, electrocardiogram; ECHO, echocardiogram; IHC, immunohistochemistry; Len, lenvatinib; LVEF, left ventricular ejection fraction; Pembro, pembrolizumab; pMMR, mismatch-repair proficient; QD, once daily; Q3W, every 3 weeks.

Given the characterization of key ARs associated with lenvatinib plus pembrolizumab described above, we offer an iterative vignette based on real cases observed in the clinic (Fig. 5) to provide an example for how a patient may be managed to maximize treatment benefit and minimize the need for treatment discontinuation. The clinical team screened and prepared the patient ensuring adequately controlled BP, urine protein levels, and cardiac function. Subspecialist consultations (cardiology, nutrition, gastrointestinal) were recommended at various points during treatment as needed, ensuring comprehensive management and optimizing the probability of quick resolution or improvement of ARs. Relatedness to either treatment was determined when possible and guidelines per the labels and study protocol were followed by the multidisciplinary team, thus enabling the patient to continue treatment (Fig. 5).

## Discussion

Tyrosine kinase inhibitors are associated with ARs such as hypertension, fatigue, nausea, and diarrhea, across a variety of indications,^10,17-19^ and immune checkpoint inhibitors are associated with imARs such as pneumonitis, colitis, and hepatitis.^12,20-22^ To maximize patients’ benefits with a combination therapy such as lenvatinib plus pembrolizumab, healthcare providers should familiarize themselves with the management strategies for ARs associated with each monotherapy and the combination. AR management approaches include patient and treatment-team education, preventative monitoring, identification of therapy-related events, and dose modifications and/or concomitant medications as needed.

The efforts required from the multidisciplinary team including nurses, nurse practitioners and physician assistants, physicians, and pharmacists, for the appropriate and prompt management of ARs, cannot be overstated. Equally important is keeping patients and caregivers informed and maintaining a shared decision strategy. Prior to beginning treatment, we recommend proactive training of nurses, nurse practitioners, and physician assistants in particular, given that they are often the first or most frequent points of contact with the patient and/or caregivers.

The treatment benefit of lenvatinib plus pembrolizumab in Study-309/KEYNOTE-775 was observed throughout the study despite patients undergoing study dose modifications^16^; data from clinical trials across tumor types support the rationale to start lenvatinib at the recommended starting dose with reduction or interruption as necessary.^13,23-25^ Optimal medical management should be used and lenvatinib and/or pembrolizumab dose interruptions or lenvatinib dose reductions initiated according to the respective prescribing information.

We recommend reviewing concomitant medications at follow-up visits and evaluating patients for overlapping toxicities that can arise from either lenvatinib or pembrolizumab.

In Study-309/KEYNOTE-775, ARs recorded with the combination of lenvatinib plus pembrolizumab first occurred within approximately 3 months of treatment initiation for all patients and the pMMR population. ARs with the shortest median time to onset (<4 weeks) included hypertension, fatigue, and musculoskeletal disorders. ARs with a relatively longer time to onset (>8 weeks) included hypothyroidism, PPES, and weight decreased. As the majority of ARs arise in the initial phase of treatment, weekly clinical assessments during the initial 2-3 cycles of therapy can afford the ability to promptly manage ARs. Attention to baseline comorbidities that may necessitate optimization prior to treatment, followed by diligent on-treatment monitoring of patients based on the prescribing information and guidelines, is crucial as ARs can occur at any time during treatment.

Establishing a culture of shared responsibility, where patients are encouraged to be proactive regarding prompt AR notification, is highly beneficial in addressing toxicity at lower grades before they escalate and allowing early initiation of supportive care and subspeciality consultation as needed for maximizing the potential for clinical benefit.

## Conclusions

In Study-309/KEYNOTE-775, successful AR management strategies included educating and preparing the patients, preventative measures, monitoring, dose modifications, and concomitant medications. The safety profile of lenvatinib plus pembrolizumab was generally consistent with that of each monotherapy and of the combination in patients with EC and other solid tumor types. The clinical team plays a critical role in prompt identification and management of ARs and should follow AR-management guidelines from the respective product labels to improve tolerance for the combination with the aim of maximizing efficacy while prioritizing safety and quality of life for patients.

## Supplementary Material

oyad201_suppl_Supplementary_Material

## Data Availability

The data will not be available for sharing at this time because the data are commercially confidential. However, Eisai Inc. will consider written requests to share the data on a case-by-case basis. N. Colombo (lead/corresponding author) confirms that she had full access to all the data in the study and takes responsibility for the integrity of the data and the accuracy of the data analysis.
