# Supplementary material for: Characterization and Management of Adverse Reactions in Patients With Advanced Endometrial Cancer Receiving Lenvatinib Plus Pembrolizumab
Source: Oncologist. 2023 Jul 31;29(1):25–35. doi: 10.1093/oncolo/oyad201 (PMC10769802; doi:10.1093/oncolo/oyad201)

Supplementary Table 1. Selected adverse-event grades according to CTCAE v4.03

	Grade 1	Grade 2	Grade 3	Grade 4
**Hypertension**	• Systolic BP 120−139 mm Hg or diastolic BP 80−89 mm Hg	• Systolic BP 140−159 mm Hg or diastolic BP 90−99 mm Hg<br>• Medical intervention indicated<br>• Recurrent or persistent (≥24 hrs)<br>• Symptomatic increase by >20 mm Hg (diastolic) or to >140/90 mm Hg<br>• Monotherapy indicated	• Systolic BP ≥160 mm Hg or diastolic BP ≥100 mm Hg<br>• Medical intervention indicated<br>• More than one drug or more intensive therapy than previously used indicated[a]	• Life-threatening consequences (eg, malignant hypertension, transient or permanent neurologic deficit, hypertensive crisis)<br>• Urgent intervention indicated
**Fatigue**	• Fatigue relieved by rest	• Fatigue not relieved by rest<br>• Limiting instrumental ADL	• Fatigue not relieved by rest<br>• Limiting self-care ADL	
**Nausea**	• Loss of appetite without alteration in eating habits	• Oral Intake decreased without significant weight loss, dehydration, or nutrition	• Inadequate oral caloric or fluid intake<br>• Tube feeding or hospitalization indicated	
**Vomiting**	• 1−2 episodes (separated by 5 minutes) in 24 h	• 3–5 episodes (separated by 5 minutes) in 24 h	• ≥6 episodes (separated by 5 minutes) in 24 h<br>• Tube feeding, TPN, or hospitalization indicated	• Life-threatening consequences<br>• Urgent intervention indicated
**Diarrhea**	• Increase of <4 stools per day over baseline<br>• Mild increase in ostomy output compared with baseline	• Increase of 4–6 stools per day over baseline<br>• Moderate increase in ostomy output compared with baseline	• Increase of ≥7 stools per day over baseline<br>• Incontinence<br>• Hospitalization indicated<br>• Severe increase in ostomy output compared with baseline<br>• Limiting self care ADL	• Life-threatening consequences<br>• Urgent intervention indicated
**Anorexia[b] (decreased appetite)**	• Loss of appetite without alteration in eating habits	• Oral intake altered without significant weight loss or malnutrition<br>• Oral nutritional supplements indicated	• Associated with significant weight loss or malnutrition (eg, inadequate oral caloric and/or fluid intake)<br>• Tube feeding or TPN indicated	• Life-threatening consequences<br>• Urgent intervention indicated
**Weight loss**	• 5 to <10% loss from baseline<br>• Intervention not indicated	• 10 to <20% loss from baseline<br>• Nutritional support indicated	• ≥20% loss from baseline<br>• Tube feeding or TPN indicated	

**Hypothyroidism**	• Asymptomatic<br>• Clinical or diagnostic observations only<br>• Intervention not indicated	• Symptomatic<br>• Thyroid replacement indicated<br>• Limiting instrumental ADL	• Severe symptoms<br>• Limiting self care ADL<br>• Hospitalization indicated	• Life-threatening consequences<br>• Urgent intervention indicated
**PPES**	• Minimal skin changes or dermatitis (eg, erythema, edema, or hyperkeratosis) without pain	• Skin changes (eg, peeling, blisters, bleeding, fissures, edema, or hyperkeratosis) with pain<br>• Limiting instrumental ADL	• Severe skin changes (eg, peeling, blisters, bleeding, fissures, edema, or hyperkeratosis) with pain<br>• Limiting self care ADL	
**Musculoskeletal pain**	• Mild pain	• Moderate pain<br>• Limiting instrumental ADL	• Severe pain<br>• Limiting self care ADL	
**Oral mucositis[b] (Stomatitis)**	• Asymptomatic or mild symptoms<br>• Intervention not indicated	• Moderate pain; not interfering with oral intake<br>• Modified diet indicated	• Severe pain; interfering with oral intake	• Life-threatening consequences<br>• Urgent intervention indicated
**Proteinuria**	• 1+ proteinuria; urinary protein <1.0 g/24 h	• 2+ proteinuria; urinary protein 1.0−3.4 g/24 h	• Urinary protein ≥3.5 g/24 h	

Reference: CTCAE version 4.03, published June 14, 2010. https://evs.nci.nih.gov/ftp1/CTCAE/CTCAE_4.03/CTCAE_4.03_2010-06-14_QuickReference_8.5x11.pdf

[a]These criteria were not used in Study-309/KEYNOTE-775. Hypertension was assessed by BP only.

[b]Specific CTCAE grading is not available for the preferred term. The preferred term used in this analysis is included in parentheses.

ADL, activities of daily living; BP, blood pressure; CTCAE, Common Terminology Criteria for Adverse Events; PPES, palmar-plantar erythrodysesthesia syndrome; TPN, total parenteral nutrition.

Supplementary Table 2. Exposure-Adjusted Incidence of Key Adverse Reactions in Study-309/KEYNOTE-775 (Safety Analysis Population)

Characteristic	pMMR Population<br><br>Lenvatinib + Pembrolizumab	All Patients<br><br>Lenvatinib + Pembrolizumab
**Patients exposed, n**	342	406
**Total exposure[a], person-months**	3174.3	3919.5
**Adverse Reactions, Event Count (Event Rate [b])**		
**Diarrhea**	443 (14.0)	525 (13.4)
**Hypertension**	386 (12.2)	448 (11.4)
**Musculoskeletal disorders**	380 (12.0)	448 (11.4)
**Hypothyroidism**	276 (8.7)	342 (8.7)
**Fatigue**	271 (8.5)	323 (8.2)
**Nausea**	257 (8.1)	306 (7.8)
**Vomiting**	249 (7.8)	297 (7.6)
**Decreased appetite**	199 (6.3)	239 (6.1)
**Proteinuria**	163 (5.1)	205 (5.2)
**Stomatitis**	162 (5.1)	200 (5.1)
**Weight decreased**	131 (4.1)	159 (4.1)
**PPES**	89 (2.8)	101 (2.6)

[a]Drug exposure is defined as the interval between the first dose date + 1 day and the earlier of the last dose date + 30 or the database cutoff date.
[b]Event rate = (event count/total exposure person-months)*100
pMMR, mismatch repair-proficient; PPES, palmar-plantar erythrodysesthesia syndrome.

Supplementary Table 3. Summary of Concomitant Medications for the Management of Key Adverse Reactions in the pMMR Population From Study-309/KEYNOTE-775 (Safety Analysis Population)

**Adverse Reaction**<br>Medications received[a], n[b] (%)	**All Patients; Lenvatinib + Pembrolizumab Group (n = 342)**
**Hypothyroidism**	
**Patients with this AR**	**229 (100.0)**
**Patients who received ≥ 1 concomitant medication**	**180 (78.6)**
Levothyroxine sodium	177 (77.3)
**Hypertension**	
**Patients with this AR**	**228 (100.0)**
**Patients who received ≥ 1 concomitant medication**	**186 (81.6)**
Amlodipine	70 (30.7)
Amlodipine besilate	41 (18.0)
Losartan	24 (10.5)
Captopril	16 (7.0)
Ramipril	19 (8.3)
Furosemide	15 (6.6)
Nifedipine	15 (6.6)
Hydrochlorothiazide	14 (6.1)
Lisinoprol	12 (5.3)
**Fatigue**	
**Patients with this AR**	**198 (100.0)**
**Patients who received ≥ 1 concomitant medication**	**10 (5.1)**
Dexamethasone	4 (2.0)
**Diarrhea[c]**	
**Patients with this AR**	**188 (100.0)**
**Patients who received ≥ 1 concomitant medication**	**121 (64.4)**
Loperamide hydrochloride	51 (27.1)
Loperamide	50 (26.6)
**Musculoskeletal disorders**	
**Patients with AR**	**181 (100.0)**
**Patients who received ≥ 1 concomitant medication**	**105 (58.0)**
Paracetamol	50 (27.6)
Ibuprofen	23 (12.7)
Loxoprofen sodium	12 (6.6)
Prednisone	9 (5.0)
**Nausea**	
**Patients with this AR**	**169 (100.0)**
**Patients who received ≥ 1 concomitant medication**	**111 (65.7)**
Ondansetron	39 (23.1)
Metoclopramide hydrochloride	31 (18.3)
Metoclopramide	24 (14.2)
Prochlorperazine	13 (7.7)
**Decreased appetite**	
**Patients with this AR**	**152 (100.0)**
**Patients who received ≥ 1 concomitant medication**	**36 (23.7)**

Megestrol acetate	8 (5.3)
Nutrients not otherwise specified	8 (5.3)
**Vomiting**	
**Patients with this AR**	**125 (100.0)**
**Patients who received ≥ 1 concomitant medication**	**45 (36.0)**
Metoclopramide	13 (10.4)
Ondansetron	13 (10.4)
Metoclopramide hydrochloride	11 (8.8)
**Stomatitis**	
**Patients with this AR**	**120 (100.0)**
**Patients who received ≥ 1 concomitant medication**	**76 (63.3)**
Nystatin	15 (12.5)
Dexamethasone	9 (7.5)
Sodium gualenate	9 (7.5)
Chlorhexidine gluconate	8 (6.7)
Lidocaine	6 (5.0)
**Weight loss**	
**Patients with this AR**	**117 (100.0)**
**Patients who received ≥ 1 concomitant medication**	**12 (10.3)**
Nutrients not otherwise specified	4 (3.4)
**Proteinuria**	
**Patients with this AR**	**100 (100.0)**
**Patients who received ≥ 1 concomitant medication**	**5 (5.0)**
Akritoin	1 (1.0)
Ciprofloxacin hydrochloride	1 (1.0)
Levothyroxine sodium	1 (1.0)
Losartan potassium	1 (1.0)
Olmesartan	1 (1.0)
Pantoprazole	1 (1.0)
Trimethoprim	1 (1.0)
**Palmar-plantar erythrodysesthesia syndrome**	
**Patients with this AR**	**77 (100.0)**
**Patients who received ≥ 1 concomitant medication**	**53 (68.8)**
Clobetasol propionate	14 (18.2)
Urea	8 (10.4)
Mucopolysaccharide polysulfuric acid ester	6 (7.8)
Heparinoid	5 (6.5)
Difluprednate	4 (5.2)
Paracetamol	4 (5.2)

[a]Medications included are those received in ≥ 5% of patients or the most common concomitant medication for the listed adverse reaction. [b]Patients may have received more than 1 medication to treat a specific adverse reaction.
pMMR, mismatch repair-proficient.

Supplementary Figure 1. Progression-free Survival[a] by Histology: (A) in pMMR Patients and (B) All Patients From Study-309/KEYNOTE-775

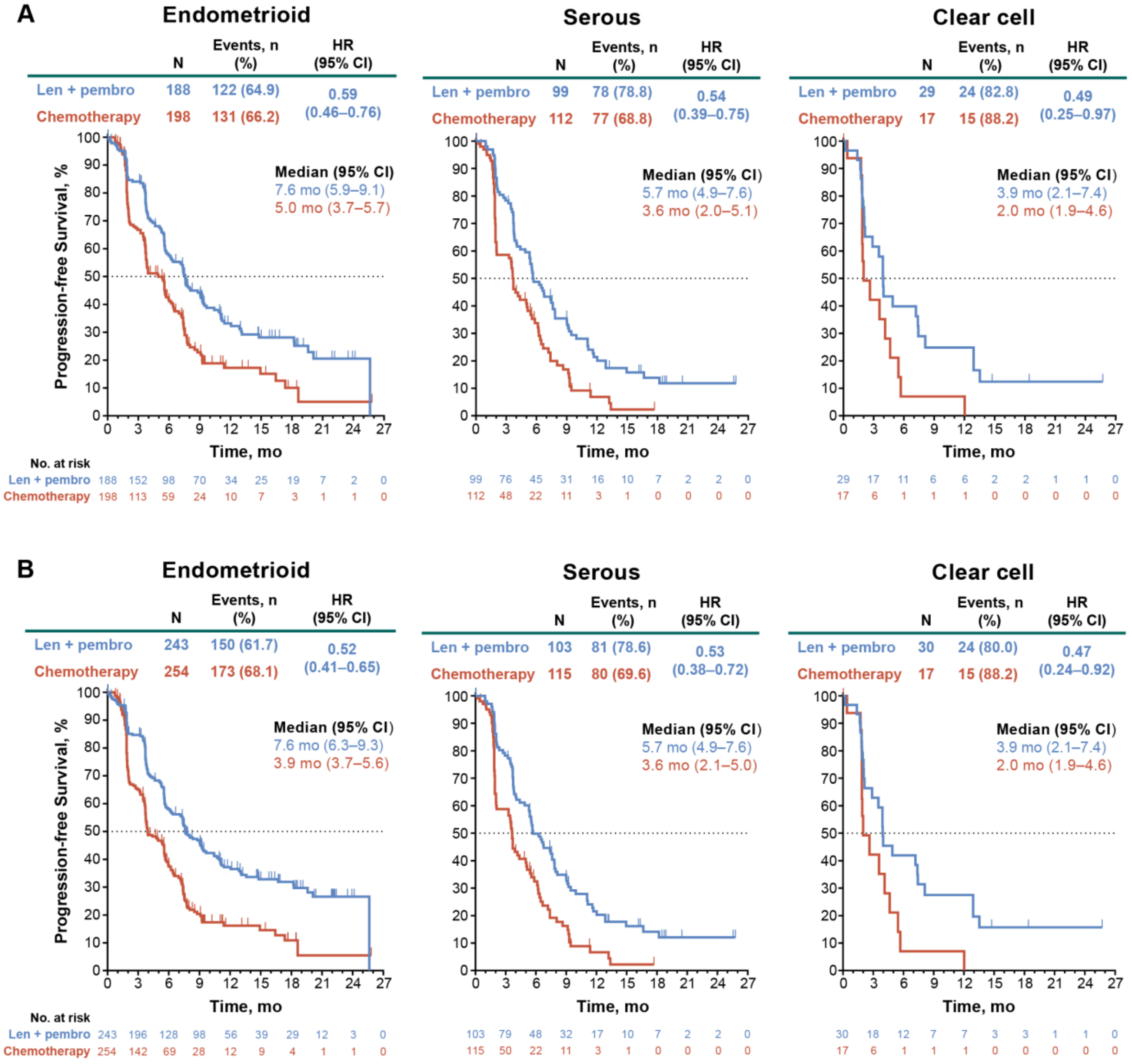

[a]Per RECIST v1.1 by BICR. Randomization was stratified by MMR status.
HR (95% CI) for other histologic types in the pMMR population: mixed cell (n = 31): 0.90 (0.35-2.29); other (n = 23): 0.38 (0.12-1.19). HR (95% CI) for other histologic types in all patients: mixed cell (n = 38): 0.90 (0.38-2.17); other (n = 27): 0.57 (0.21-1.54).
Data cutoff date: October 26, 2020.

BICR, blinded independent central review; CI, confidence interval; HR, hazard ratio; Len, lenvatinib; MMR, mismatch repair; pembro, pembrolizumab; pMMR, mismatch-repair proficient; RECIST v1.1; Response Evaluation Criteria in Solid Tumors version 1.1.

Supplementary Figure 2. Overall Survival by Histology: (A) in pMMR Patients and (B) All Patients From Study-309/KEYNOTE-775

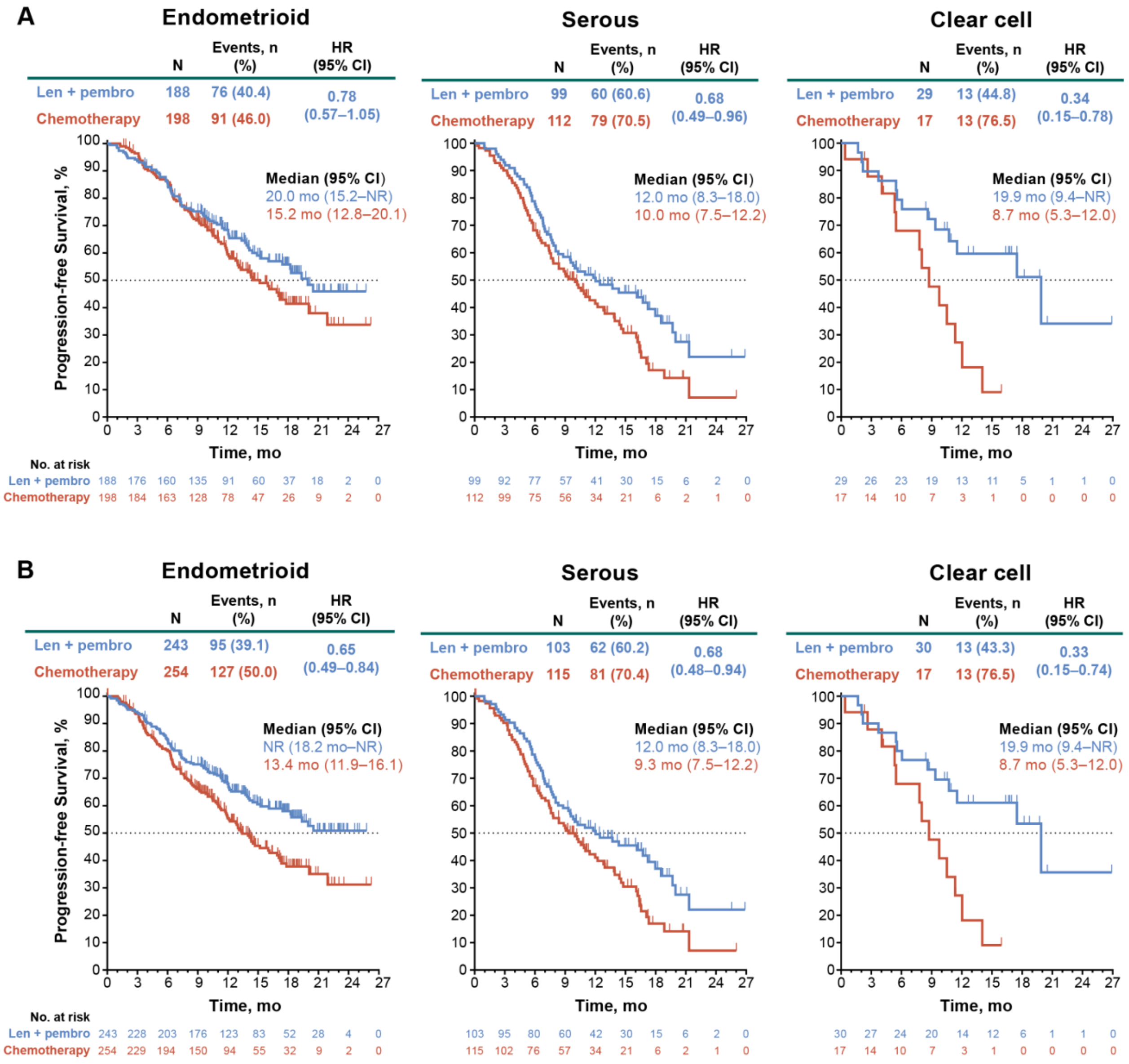

HRs for other histologic types in the pMMR population: mixed cell (n = 31): HR (95% CI), 0.40 (0.17-0.99); other (n = 23): HR (95% CI), 0.32 (0.11-0.94). HRs for other histologic types in all patients: mixed cell (n = 38): HR (95% CI), 0.37 (0.16-0.85); other (n = 27): HR (95% CI), 0.39 (0.15-1.04).

BICR, blinded independent central review; CI, confidence interval; HR, hazard ratio; Len, lenvatinib; MMR, mismatch repair; pembro, pembrolizumab; pMMR, mismatch-repair proficient; RECIST v1.1; Response Evaluation Criteria in Solid Tumors version 1.1.

Supplementary Figure 3. (A) Progression-free Survival[a] and (B) Overall Survival by Prior Therapy in pMMR Patients and All Patients From Study-309/KEYNOTE-775

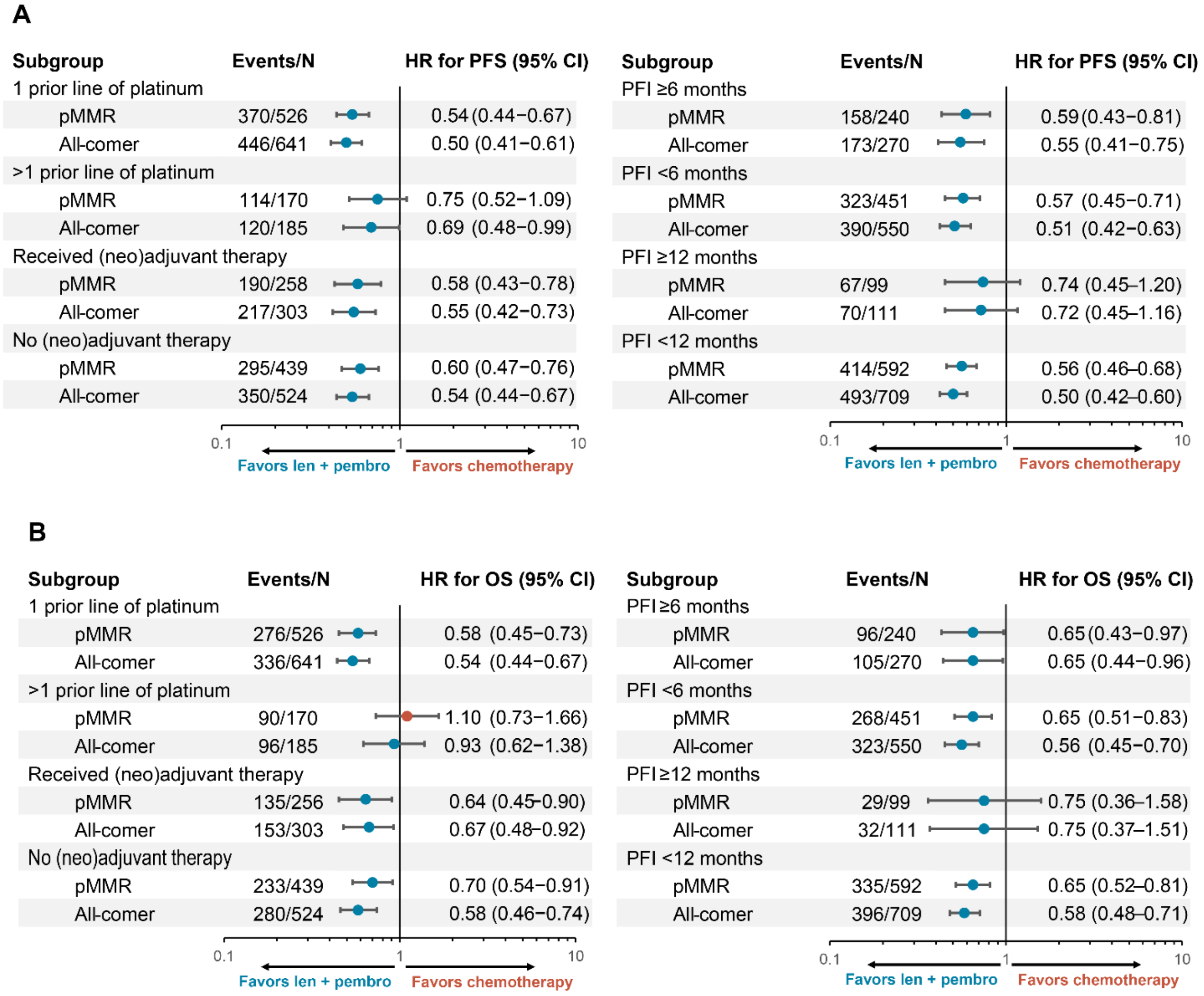

[a]Per RECIST v1.1 by BICR.

BICR, blinded independent central review; CI, confidence interval; HR, hazard ratio; len, lenvatinib; MMR, mismatch repair; OS, overall survival; pembro, pembrolizumab; PFI, platinum-free interval from most recent platinum-containing regimen; PFS, progression-free survival; pMMR, mismatch-repair proficient; RECIST v1.1; Response Evaluation Criteria in Solid Tumors version 1.1.

Supplementary Figure 4. Subsequent Systemic Anticancer Therapies Received by (A) Treatment Type and by (B) Treatment and Line of Therapy

A

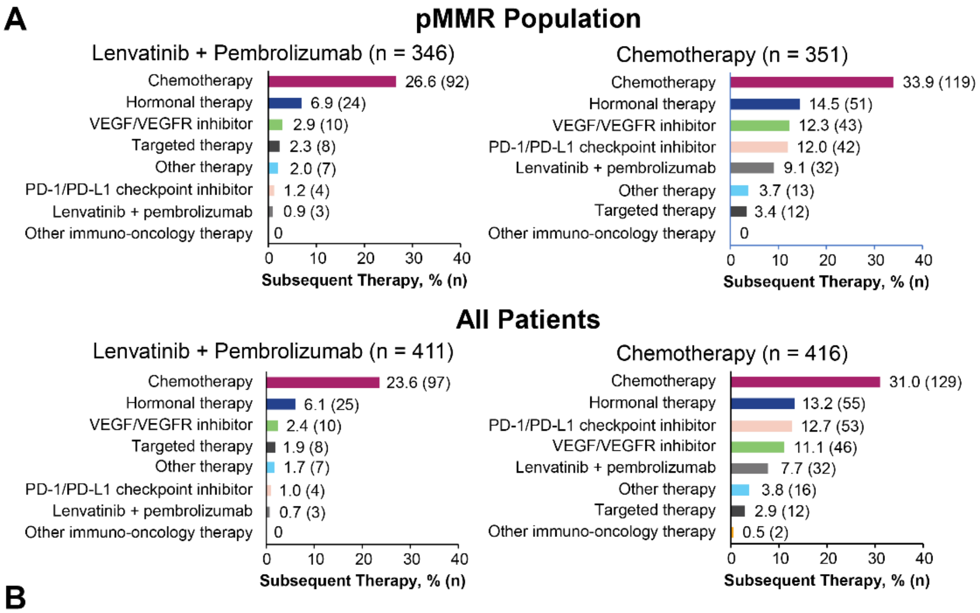

B

Subsequent Anticancer Therapies, n (%)	pMMR Population		All Patients		Total (N = 827)
	Lenvatinib + Pembrolizumab (n = 346)	Chemotherapy (n = 351)	Lenvatinib + Pembrolizumab (n = 411)	Chemotherapy (n = 416)	
Any subsequent anticancer therapy	109 (31.5)	176 (50.1)	115 (28.0)	200 (48.1)	315 (38.1)
Subsequent anticancer therapies received in ≥ 5% of all patients					
Paclitaxel	33 (9.5)	50 (14.2)	35 (8.5)	57 (13.7)	92 (11.1)
Carboplatin	30 (8.7)	47 (13.4)	30 (7.3)	52 (12.5)	82 (9.9)
Doxorubicin	55 (15.9)	16 (4.6)	58 (14.1)	18 (4.3)	76 (9.2)
Gemcitabine	14 (4.0)	34 (9.7)	15 (3.6)	35 (8.4)	50 (6.0)
Pembrolizumab	4 (1.2)	38 (10.8)	4 (1.0)	46 (11.1)	50 (6.0)
Subsequent lenvatinib + pembrolizumab	3 (0.9)	32 (9.1)	3 (0.7)	32 (7.7)	35 (4.2)

Percentages are out of all patients, regardless of whether subsequent therapy was received; patients may have received > 1 subsequent therapy regimen.

PD-1/PD-L1, programmed cell death/ programmed cell death ligand 1; pMMR, mismatch repair-proficient; VEGF/VEGFR, vascular endothelial growth factor/vascular endothelial growth factor receptor.

Supplementary Figure 5. Kaplan–Meier Curves of PFS2 in the (A) pMMR population and (B) All Patients

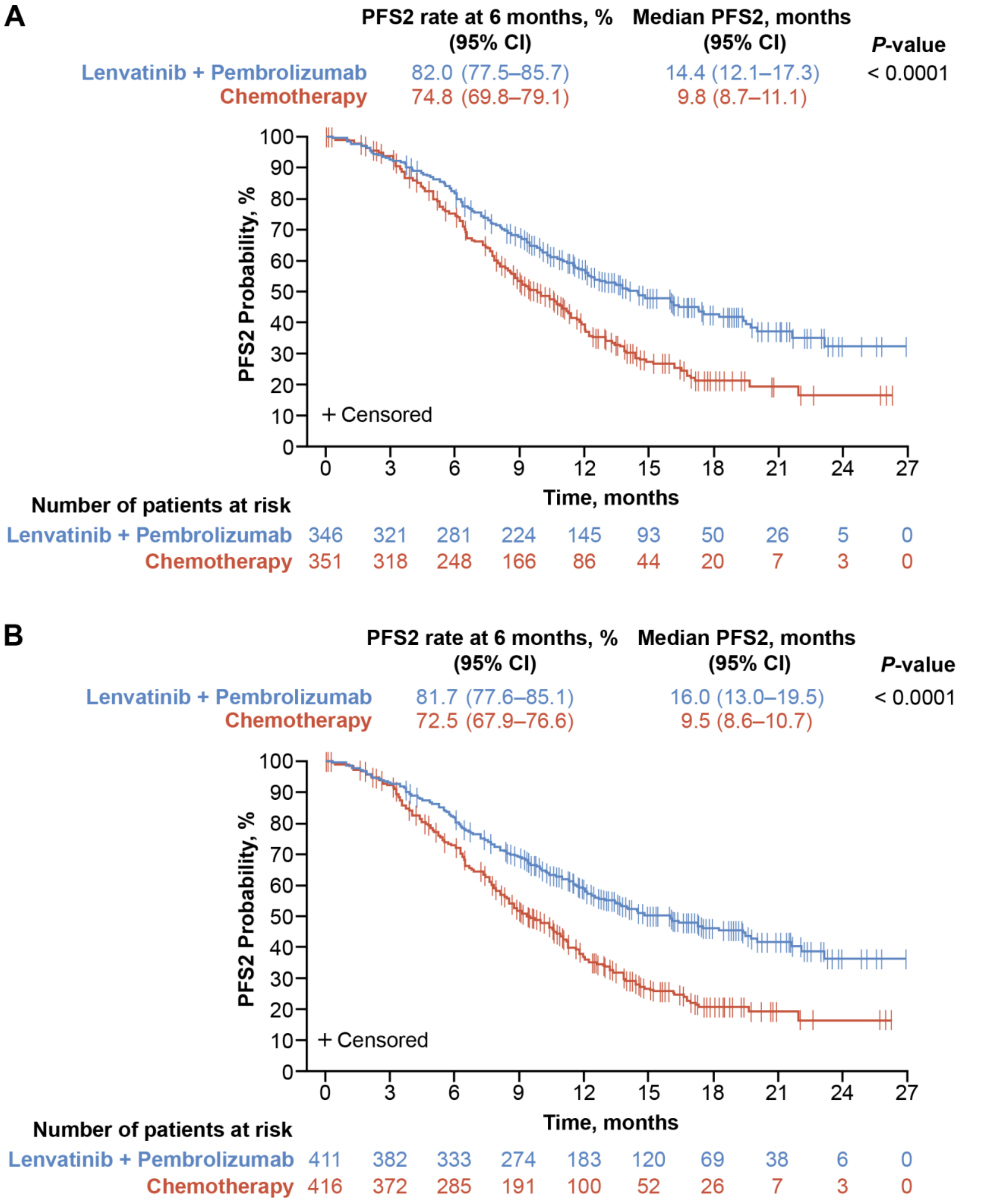

CI, confidence interval; PFS2, progression-free survival on next line of therapy; pMMR, mismatch repair-proficient.

Supplementary Figure 6. Median Time to First Onset[a] of Key Adverse Reactions and Dose Management in the pMMR Population From Study-309/KEYNOTE-775 (Safety Analysis Population)

pMMR Population (n = 342)

Adverse Reaction	Incidence[b] n	Incidence[b] %	Len Dose Interruption[c]	Len Dose Reduction[c]	Len Discontinuation[c]	Pembro Dose Interruption[c]	Pembro Discontinuation[c]	Median Time to First Onset (weeks)[a]
Hypertension	228	66.7%	12.3%	17.5%	2.0%	3.5%	0%	MIN: 0.1 / Q1: 0.71 / Median: 2.1 / Q3: 3.50 / MAX: 39.0 weeks
Fatigue	198	57.9%	6.1%	13.2%	2.0%	4.7%	0.3%	MIN: 0.1 / Q1: 1.00 / Median: 2.5 / Q3: 10.57 / MAX: 88.0 weeks
Musculoskeletal disorders	181	52.9%	4.1%	5.6%	0.6%	3.5%	0.3%	MIN: 0.1 / Q1: 1.00 / Median: 3.1 / Q3: 9.00 / MAX: 93.7 weeks
Proteinuria	100	29.2%	6.7%	7.0%	1.2%	2.3%	0%	MIN: 0.1 / Q1: 2.14 / Median: 3.8 / Q3: 9.36 / MAX: 76.0 weeks
Stomatitis	120	35.1%	1.8%	4.1%	0.3%	0.3%	0%	MIN: 0.1 / Q1: 1.29 / Median: 3.9 / Q3: 8.64 / MAX: 77.9 weeks
Decreased appetite	152	44.4%	5.0%	6.1%	1.5%	2.3%	0.9%	MIN: 0.1 / Q1: 1.21 / Median: 4.9 / Q3: 13.29 / MAX: 61.7 weeks
Nausea	169	49.4%	3.5%	4.4%	0.6%	1.5%	0%	MIN: 0.1 / Q1: 1.43 / Median: 5.0 / Q3: 14.29 / MAX: 67.3 weeks
Diarrhea	188	55.0%	11.1%	11.7%	1.2%	8.2%	0.9%	MIN: 0.1 / Q1: 2.43 / Median: 8.1 / Q3: 17.86 / MAX: 78.7 weeks
Vomiting	125	36.5%	5.0%	2.3%	1.2%	1.8%	0%	MIN: 0.1 / Q1: 2.71 / Median: 8.4 / Q3: 19.29 / MAX: 60.3 weeks
Hypothyroidism	229	67.0%	2.0%	0.9%	0%	1.8%	0.3%	MIN: 2.0 / Q1: 3.14 / Median: 8.4 / Q3: 14.86 / MAX: 72.3 weeks
PPES	77	22.5%	2.0%	8.8%	0.6%	2.0%	0%	MIN: 0.4 / Q1: 3.71 / Median: 10.0 / Q3: 15.86 / MAX: 77.7 weeks
Weight decreased	117	34.2%	2.6%	5.0%	0.9%	1.5%	0.3%	MIN: 0.1 / Q1: 6.14 / Median: 12.1 / Q3: 20.86 / MAX: 69.0 weeks

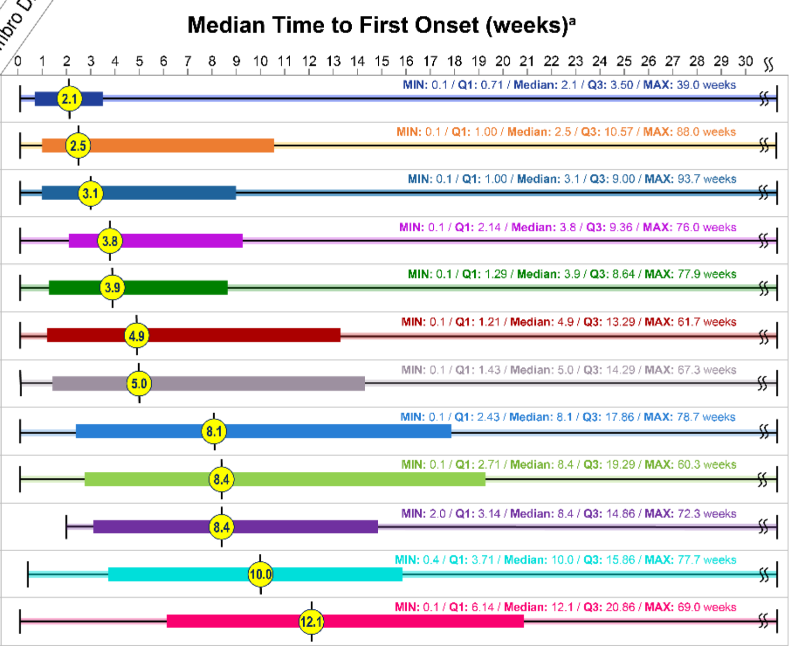

[a]Median time to first onset in patients who experienced the adverse reaction.
[b]All grades.
[c]Percentages of dose modifications and discontinuations were based on the safety analysis set.

Len, lenvatinib; max, maximum; min, minimum; Pembro, pembrolizumab; PPES, palmar-plantar erythrodysesthesia syndrome; Q, quartile.

Supplementary Figure 7. Preventative Measures, Monitoring, and Management of ARs Associated With Lenvatinib and/or Pembrolizumab During Pretreatment and Treatment Phases

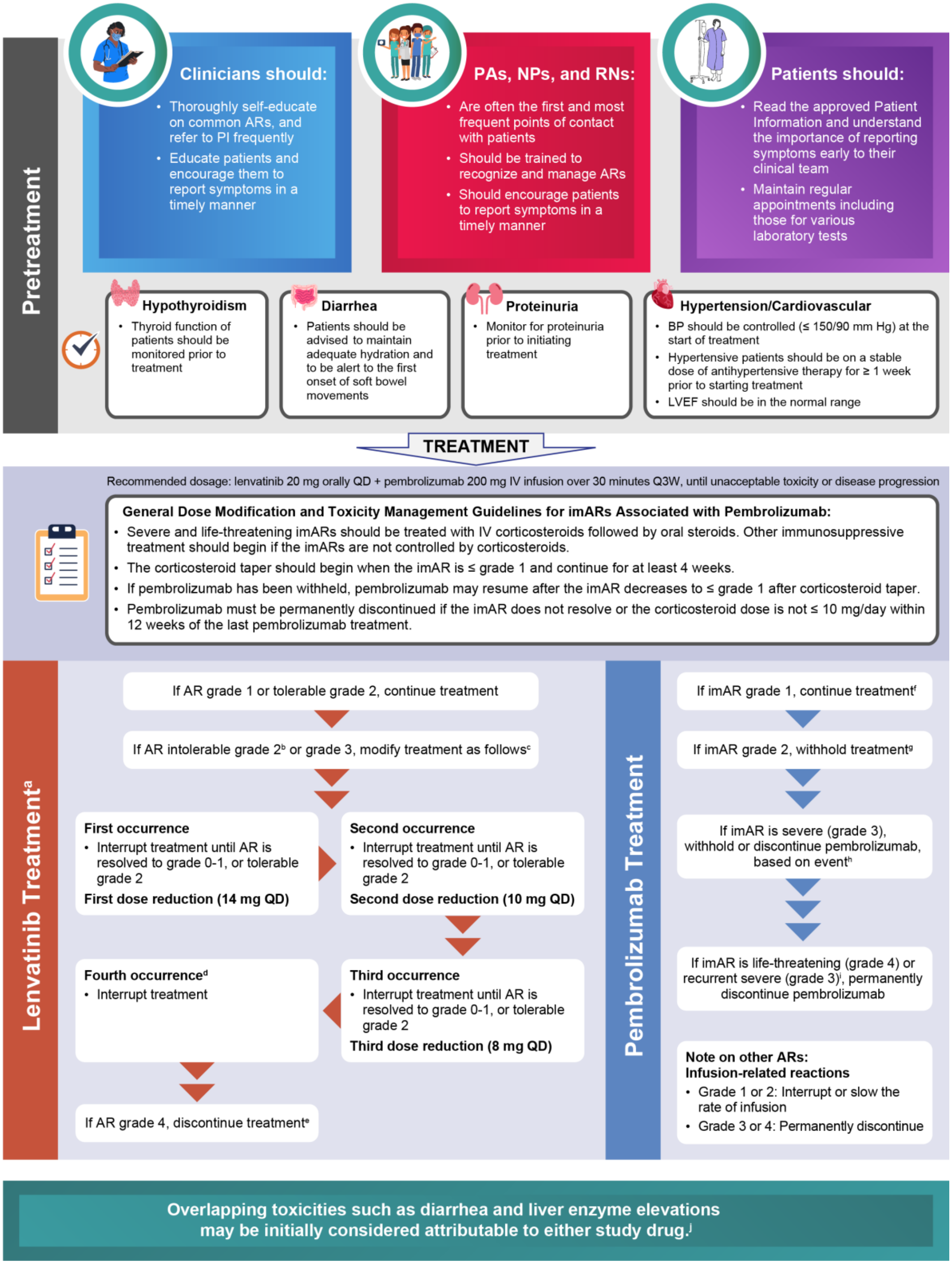

Please note there are exceptions to the grade 3- and grade 4-severity advice; some grade 3 ARs require treatment discontinuation, whereas some grade 4 ARs do not (please see the lenvatinib and pembrolizumab prescribing information for further details). In the protocol for Study-309/KEYNOTE-775, it was recommended that patients resume lenvatinib upon resolution of the AR to tolerable grade 2 or grade ≤ 1 severity. Dose reductions of pembrolizumab are not recommended.

[a]Initiate optimal medical management for nausea, vomiting, hypertension, hypothyroidism and/or diarrhea prior to any lenvatinib interruption or dose reduction.

[b]Obese patients (BMI ≥30) with weight loss do not need to return to the baseline weight or 10% of baseline weight (ie, grade 1 weight loss). These patients may restart the study drug(s) at a lower dose once their weight remains stable for at least 1 week and they have a minimum BMI of 25. The new stable weight should be used as the new baseline for further dose reductions.

[c]Per the study protocol, for asymptomatic laboratory abnormalities, such as grade ≥3 elevations of amylase and lipase that are not considered clinically relevant by the investigator, continuation of treatment without dose modification should be discussed with the clinical team.

[d]Per the study protocol, further dose reduction to 4 mg was allowed with sponsor approval.

[e]Excluding laboratory abnormalities judged to be non-life-threatening, in which case manage as grade 3.

[f]Withhold for grade 1 myocarditis.

[g]Resume in patients with complete or partial resolution (grades 0 to 1) after corticosteroid taper. Permanently discontinue if no complete or partial resolution within 12 weeks of initiating steroids or inability to reduce prednisone to 10 mg per day or less (or equivalent) within 12 weeks of initiating steroids. Permanently discontinue for grade ≥ 2 myocarditis. Per the study protocol, pembrolizumab treatment could be continued for grade 2-4 hypothyroidism or grade 2 hyperthyroidism.

[h]Events that require discontinuation include but are not limited to Guillain-Barré Syndrome, encephalitis, Stevens-Johnson Syndrome, toxic epidermal necrolysis, and grades 3 or 4 pneumonitis, alanine aminotransferase and aspartate aminotransferase elevation to more than 8 times ULN or increased total bilirubin to more than 3 times ULN, and grade 4 nephritis with renal dysfunction.

[i]Permanently discontinue pembrolizumab for recurrent severe (grade 3) immune-mediated reactions that require systemic immunosuppressive treatment, or an inability to reduce corticosteroid dose to 10 mg or less of prednisone or equivalent per day within 12 weeks of initiating steroids.

[j]See Supplementary Figure 8 for further information on management of overlapping toxicities.

AR, adverse reaction; BP, blood pressure; IV, intravenous; LVEF, left ventricular ejection fraction; PA, physician assistant; PI, prescribing information; RN, registered nurse; QD, once daily; Q3W, every 3 weeks.

Supplementary Figure 8. Management Strategies for Overlapping Toxicities

General Dose Modification Guidelines for Overlapping Toxicities:

Timing of AR onset

Lenvatinib is dosed daily and continuously due to a relatively short half-life (28 hours), and pembrolizumab is dosed Q3W due to a long half-life.

- If an AR is identified during a treatment cycle (ie, between 2 pembrolizumab doses), only lenvatinib dose interruption is needed.
- If an AR is identified at the beginning of a treatment cycle, lenvatinib can be interrupted, and dosing of pembrolizumab should be held.

If the patient recovers from an AR in response to lenvatinib interruption (ie, positive dechallenge), the event is more likely to be related to lenvatinib. Otherwise, after excluding other alternative explanations, an immune-related AR should be considered.

Severity of AR

If an AR is suspected to be treatment-related and is severe/life-threatening at the time of onset or is rapidly worsening, interruption of both drugs and treatment with a corticosteriod (with exception of hypothyroidism, TIDM) and other supportive care should be initiated promptly.

The median times to onset and resolution of ARs are important factors to help determine relative causality association with treatment.

AR, adverse reaction; Q3W, every 3 weeks; TIDM, type 1 diabetes mellitus.

Supplementary Appendix

Chemotherapy regimens included in Study-309/KEYNOTE-775

The following chemotherapy regimens of physicians' choice were included in Study-309/KEYNOTE-775: doxorubicin 60 mg/m^2 of body-surface area, intravenously as a 1-hour infusion or according to institutional guidelines, every 3 weeks, *or* paclitaxel at 80 mg/m^2 intravenously as a 1-hour infusion or according to institutional guidelines, weekly (with a cycle of 3 weeks on and 1 week off) (Makker 2022).

Definition of adverse reaction (AR)

To aid patient management decisions, the Food and Drug Administration (FDA) pools adverse events considered reasonably similar into grouped terms called ARs (FDA AR guidance). ARs are adverse events that are considered reasonably associated with the use of a drug, although a definitive causal relationship may not be established (FDA AR guidance). Adverse events reported under different terms but representing the same phenomenon are grouped together as a single AR by the FDA to maintain consistent and accurate reporting without introducing bias (FDA AR guidance). Key ARs were chosen regardless of causality and based on frequency of occurrence and those leading to dose reductions, interruptions, or discontinuation of study treatment.

Limitations of database in Study-309/KEYNOTE-775

Of note, a limitation of the database was that only the last action taken (interruption/reduction) was captured. This explains why the number of dose reductions recorded were at times higher than the number of dose interruptions.

Exposure-adjusted ARs

Exposure-adjusted incidences of ARs were calculated by dividing the total number of episodes (n) by total exposure (n/total exposure). Total exposure referred to the total duration of treatment (in years) among all patients.

References:

Makker V, Colombo N, Casado Herráez A, et al. Lenvatinib plus Pembrolizumab for Advanced Endometrial Cancer. N Engl J Med. 2022;386(5):437-448. doi:10.1056/NEJMoa2108330

FDA adverse reaction section guidance. https://www.fda.gov/media/72139/download

Graphical abstract:

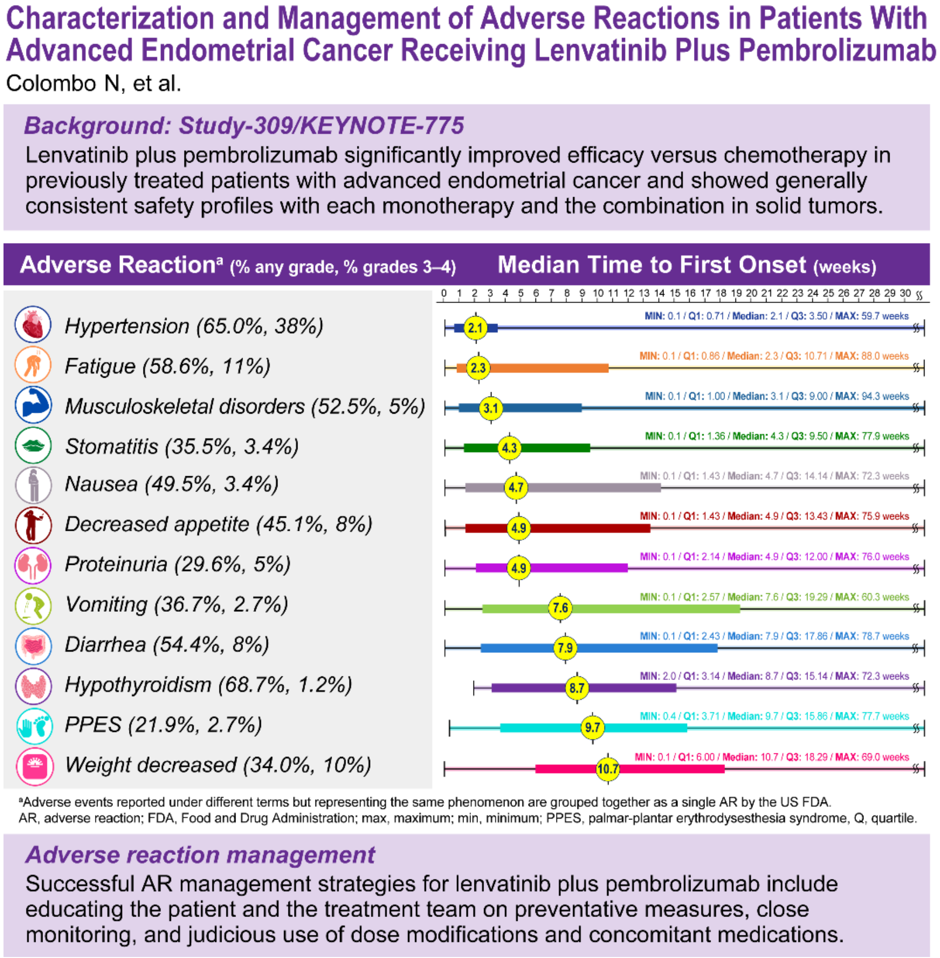

Supplement: oyad201_suppl_Supplementary_Material [file oyad201_suppl_supplementary_material.pdf]
